# Resolution of maternal Mirror syndrome after succesful fetal intrauterine therapy: a case series

**DOI:** 10.1186/s12884-018-1718-0

**Published:** 2018-04-06

**Authors:** Angel Chimenea, Lutgardo García-Díaz, Ana María Calderón, María Moreno-De Las Heras, Guillermo Antiñolo

**Affiliations:** 1 Department of Genetics, Reproduction, and Fetal Medicine, Institute of Biomedicine of Seville (IBIS), Hospital Universitario Virgen del Rocio/CSIC/University of Seville, Seville, Spain; 2 Centre for Biomedical Network Research on Rare Diseases (CIBERER), Seville, Spain

**Keywords:** Mirror syndrome, Ballantyne syndrome, Parvovirus B19, Hydrops fetalis, Fetal therapy

## Abstract

**Background:**

Mirror syndrome (MS) is a rare obstetric condition usually defined as the development of maternal edema in association with fetal hydrops. The pathogenesis of MS remains unclear and may be misdiagnosed as pre-eclampsia.

**Case presentation:**

We report a case series of MS in which fetal therapy (intrauterine blood transfusion and pleuroamniotic shunt) resulted in fetal as well as maternal favourable course with complete resolution of the condition in both mother and fetus.

**Conclusions:**

Our case series add new evidence to support that early diagnosis of MS followed by fetal therapy and clinical maternal support are critical for a good outcome.

## Background

Mirror syndrome (MS) is a rare complication of fetal hydrops appearing as a triple edema (fetal, placental as well as maternal) [1], in which the mother “mirrors” the hydropic fetus. This syndrome was first described in 1892 by the Scottish obstetrician John William Ballantyne [2].

There have been multiple feto-placental diseases related to MS, that can be classified into diverse groups based on different etiologies [3]: cardiac failure associated with fetal anemia (e.g. Parvovirus B19 [4], erytroblastosis [5], fetal alloimmune thrombocytopenia [6], hemoglobin Bart’s disease [7]); high-output cardiac failure associated with shunting (e.g. chorioangioma [8], twin to twin transfusion syndrome [9–13]); and fetal anomalies (e.g. fetal arrhythmias [14], CHAOS syndrome [15]).

MS maternal clinical picture includes peripheral edema, uterine distension and rapid weight gain [3]. Those nonspecific clinical features may lead to a misdiagnosis of pre-eclampsia, delaying diagnosis and therapy of MS and worsening fetal and maternal condition. MS does not usually present with hypertension, but blood pressure can be elevated and proteinuria can appear, resembling the clinical features of pre-eclampsia [3, 16]. Like in pre-eclampsia, laboratory findings may include proteinuria, low platelet count and elevation of creatinine, hepatic enzymes and uric acid levels [3].

The treatment of choice for MS is the resolution of the fetal hydrops. If correction of the fetal condition is not possible, delivery usually results in a favourable maternal outcome [17]. Vaginal delivery may be preferred, but complications as maternal pulmonary edema or deterioration of the fetal condition can lead to an emergent caesarean section [18–20].

## Case presentation

### Case 1

A 31-year-old G1P0 caucasian woman was referred to our centre at 27 + 2 gestational weeks for evaluation of fetal hydrops. Her medical and obstetric record as well as the previous fetal ultrasound at 20 weeks were unremarkable.

On admission the patient was normotensive, with stable vital signs. Physical examination showed a significant edema in both lower extremities and sacrum. Blood analysis results revealed a hemoglobin of 105 g/L, and a hematocrit of 28.8% with normal platelet count. Liver function test was abnormal: ALT 68 mU/ml (*N* < 40 mU/ml), AST 44 mU/ml (*N* < 37 mU/ml). LDH was elevated (240 UI/L; *N* < 225 UI/L) as well as uric acid levels (8.4 mg/dL; *N* < 7 mg/dL). Renal function was normal with a creatinine of 0.87 mg/dL (*N* < 1.1 mg/dL) and a urea of 39 mg/dL (N < 40 mg/dL). Her 24-h urinary protein excretion was 2925 mg.

**Fig. 1:**
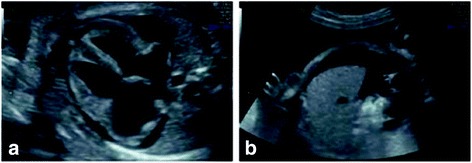
Case 1. GA: 27 + 3 weeks. Pleural effusion (**a**), ascites (**b**)

Ultrasound revealed a single fetus with hydrops including severe ascites, pericardial effusion, subcutaneous tissue edema, a slightly thickened placenta (Figure 1), and normal amniotic fluid index (AFI). No fetal anomalies were detected. Middle cerebral artery peak systolic velocity (MCA-PSV) value was 77.5 cm/s (2.19 MoM [21, 22]) with an estimated hemoglobin of 38.6 g/L (0.31 MoM [22]). The EFW was 1.073 g. Initial evaluation of fetal anemia included indirect Coomb’s test, serology for Parvovirus B19 and TORCH. Fetus was diagnosed as having a hydrops related to severe anemia and a MS was diagnosed.

Forty-eight hours following patient admission, intrauterine blood transfusion (IBT) was performed as described elsewhere [8]. Before the procedure, a single course of corticosteroid therapy was administered to accelerate fetal pulmonary maturity, and tocolysis with atosiban was started. During the intervention, fetal blood samples for genetic, anemia and infection studies were obtained, and a Kleihauer-Betke test was requested. Following IBT, fetal hemoglobin increased from 43 g/L to 109 g/L, and fetal hematocrit from 14% to 34%. Anti-D Ig 300 μg IM was administered after transfusion to prevent Rh(D) alloimmunization. The next day MCA-PSV value decreased to 36.9 cm/s (1.02 MoM) [21, 22]. In both maternal and fetal blood Indirect Immunofluorescence Test (IIFT) a positive IgM for Parvovirus B19 were found, confirming the etiology of the fetal anemia.

**Fig. 2:**
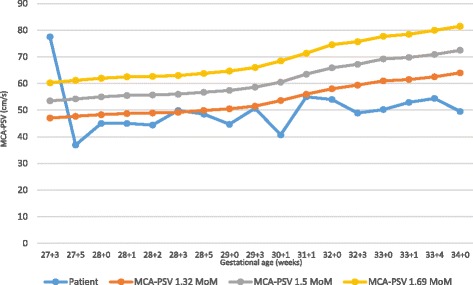
Case 1. MCA-PSV evolution. IBT was performed at 27 + 4 weeks. Blue line represents patient values. Red line represents 1.32 MoM of normal fetal MCA-PSV. Grey line represents 1.5 MoM of normal fetal MCA-PSV. Yellow line represents 1.69 MoM of normal fetal MCA-PSV

Fetal and maternal follow up revealed a progressive reduction of fetal hydrops (Figure 2) together with a progressive resolution of maternal clinical picture including normalization of laboratory tests.

At 29 + 4 gestational weeks, a preterm premature rupture of membranes occurred. At 34 gestational weeks a healthy male infant was born by vaginal delivery, with Apgar score of 8/9/10 at 1, 5 and 10 min. Both mother and child were discharched without complications and are currently doing well 2 years after delivery.

### Case 2

A 37-year-old G1P0 caucasian woman was admitted to our centre at 31 gestational weeks due to nausea, vomiting and severe abdominal pain refractory to the medical treatment. Premature labor with progressive cervical shortening was diagnosed and tocolysis with atosiban was started. Physical examination showed a significant skin edema in both lower extremities. Her vital signs and laboratory results were within normal range except for anemia (hemoglobin 89 g/L). Further laboratory test showed a marked increase in C-reactive protein to 82.3 mg/L (*N* < 5 mg/dL).

Fetal ultrasound revealed a bilateral hydrothorax with a structural and functionally normal heart and vessels. A large hyperechoic placenta and placental edema were observed as well. No fetal anomalies were detected, and AFI was in normal range. MCA-PSV was 51.9 cm/s (1.21 MoM), consistent with an estimated fetal hemoglobin of 111.1 g/L (0.85 MoM [22]).

**Fig. 3:**
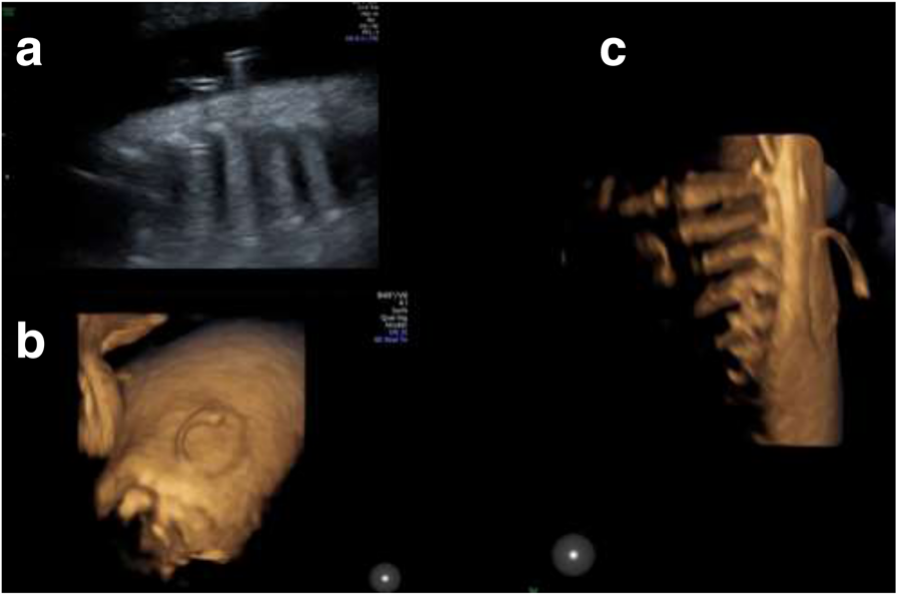
Case 2. 2-D (**a**) and 4-D (**b**, **c**) ultrasound images of the catheter pigtail correctly inserted inside and outside of the fetal hemithorax

MS was diagnosed and a pleuroamniotic shunt (rocket catheter) was placed in right hemithorax to resolve fetal hydrops. Shunting was followed by bilateral lung parenchyma expansion (Figure 3).

On post-operative day#1, a deterioration of maternal clinical status with respiratory distress associated with increasing edema and diuresis volume reduction were observed. In addition, maternal echocardiography showed evidence of moderate pericardial effusion and tricuspid insufficiency. Treatment was started with furosemide, spironolactone, seroalbumine and potassium. In the next 72 h following maternal treatment and fetal hydrops resolution, an improvement of maternal clinical condition ocurred, with progressive disappearance of maternal peripheral edema, respiratory distress and anemia.

At 35 + 2 gestational weeks preterm premature rupture of membranes occurred, followed by delivery of a healthy male newborn with Apgar score 10/10/10 at 1, 5 and 10 min. At birth no lesions in newborn costal grid related to intrauterine shunt placement were observed. Both mother and newborn were discharged without complications and are currently doing well one year after delivery.

## Discussion and conclusions

MS is a condition wherein the mother “mirrors” the edema present in the fetus. This entity was first described in association with rhesus-immunization, although MS is most commonly associated with non-immune fetal hydrops (NIHF) of an unknown etiology [19]. Anemia related to Parvovirus B19 is the most frequent reported infectious cause of NIHF. Therefore, complete serology including Parvovirus B19 is mandatory in the differential diagnosis of the fetal hydrops etiology related to anemia.

MS has many similarities to pre-eclampsia [23], sharing clinical features that may be caused in both cases by an imbalance between angiogenic and anti-angiogenic factors [24]. In pre-eclampsia there exists evidence of placental underperfusion caused by failure of trophoblastic invasion into the spiral arteries, with a subsequent increasing of circulating sFLT-1 levels and decreasing of PlGF levels [25, 26], being the second one a mechanism proposed as well as responsible for the maternal clinical findings in MS [27–31].

The association of edema, oliguria, and hemodilution might be characteristic of MS [32]. In addition, it has been suggested that the presence of hemodilution might be criteria for diagnosis of MS, as opposed to pre-eclampsia with low haematocrit [33]. MS does not usually present with oligohydramnios or hypertension [34], and unlike pre-eclampsia may be reversed if fetal hydrops is resolved. Fetal prognosis in MS is usually worse than in pre-eclampsia, resulting in many cases in intrauterine fetal demise [3], and being 56% the currently reported rate of intrauterine fetal death in MS [33].

In MS fetal hydrops successful therapy has been claimed as a key step to maternal clinical picture resolution [3, 5, 9, 10, 12, 31, 35]. According to a recent systematic review, interventions to correct the fetal hydrops related to anemia were significantly associated with improved fetal survival [35]. However, sometimes delivery is required when it is not possible to perform fetal therapy or when it is not successful [3, 12, 17, 20, 33, 36–38]. Different strategies have been described for the resolution of fetal hydrops, [3, 5, 9, 13, 14, 31, 39]. In our case series, fetal therapy (IBT and pleuroamniotic shunt) led to a slow but consistent reversal of maternal clinical picture following fetal hydrops resolution, resulting in a good fetal and maternal outcome.

Current data and experience from clinical practice support that fetal hydrops therapy, regardless etiology, improves fetal survival as well as maternal evolution in MS. The cases we described show the need for an early diagnosis of MS, followed by an adequate treatment of the fetal condition, which improves maternal condition as well as perinatal morbidity and mortality. When the underlying fetal insult is corrected, we can expect a slow but sustained recovery of maternal condition, that may require an adequate intensive support.

## Abbreviations

AFI: Amniotic Fluid Index
EFW: Estimated Fetal Weight
IBT: Intrauterine Blood Transfusion
IIFT: Indirect Immunofluorescence Test
MCA-PSV: Middle Cerebral Artery Peak Systolic Velocity
MS: Mirror Syndrome
NIHF: Non-Immune Fetal Hydrops
NST: Nonstress Test
